# Natural Bioactive Compounds in Sheep Milk: Potential Biomedical Applications

**DOI:** 10.3390/cimb47060456

**Published:** 2025-06-12

**Authors:** Zuzanna Flis, Edyta Molik, Anna Ptak, Piotr Szatkowski

**Affiliations:** 1 Department of Animal Biotechnology, Faculty of Animal Science, University of Agriculture in Krakow, Al. Mickiewicza 24/28, 31-059 Krakow, Polandedyta.molik@urk.edu.pl (E.M.); 2 Laboratory of Physiology and Toxicology of Reproduction, Institute of Zoology and Biomedical Research, Faculty of Biology, Jagiellonian University, Gronostajowa 9, 30-387 Krakow, Poland; 3 Department of Glass Technology and Amorphous Coatings, Faculty of Materials Science and Ceramics, AGH University of Krakow, Al. Mickiewicza 30, 30-059 Krakow, Poland

**Keywords:** natural products, health benefits, nutraceuticals, sheep milk, biomedicine

## Abstract

Sheep milk is a rich source of bioactive compounds with significant potential in functional foods and biomedical applications. It contains high levels of proteins, peptides, and fatty acids with numerous health-promoting properties for the human body. Key components such as lactoferrin, proline, orotic acid, and conjugated linoleic acid (CLA) support the prevention and treatment of chronic diseases such as diabetes, cardiovascular disease, obesity, cancer, and neurodegenerative disorders. Bioactive peptides from sheep milk regulate blood glucose levels by inhibiting enzymes such as dipeptidyl peptidase-IV (DPP-IV) and α-glucosidase, while conjugated linoleic acid improves lipid metabolism and reduces inflammation. The high-quality proteins in sheep milk are essential for tissue regeneration and maintaining muscle mass, which is particularly beneficial for the elderly and infants who are allergic to cow milk. Recently, there has been an increasing interest in hydrogel dressings enriched with bioactive substances from sheep milk, which support wound healing by supporting collagen synthesis, reducing inflammation, and having antimicrobial properties. Such hydrogels are particularly promising for the treatment of chronic wounds, burns, and diabetic ulcers, making them a valuable tool in regenerative medicine. The aim of this manuscript is to review the current reports on bioactive components of sheep milk and their potential for biomedical applications.

## 1. Introduction

Sheep milk has long been a subject of interest in pharmaceutical and biomedical research due to its rich composition of bioactive compounds. The bioactive components in sheep milk have the potential to modulate cell signaling pathways, influence gene expression, and interact with immune cells [1,2,3], making it a valuable resource for therapeutic applications. These substances possess various health-promoting properties, including immunomodulatory, anti-inflammatory, antioxidant, and anticancer effects [4,5,6].

Traditionally, sheep milk has been utilized in folk medicine for treating infections and promoting wound healing [7]. Recent studies have highlighted its role in supporting the relief of symptoms associated with chronic inflammation [5]. Additionally, bioactive substances in sheep milk demonstrate wound-healing, moisturizing, protective, and anti-aging effects [4], making it an attractive ingredient in therapeutic and cosmetic formulations. In modern medicine, sheep milk is incorporated into hydrogel dressings designed for the treatment of difficult-to-heal wounds, particularly in diabetic patients.

Furthermore, sheep milk is gaining recognition in the functional food market due to its high nutritional value and the presence of health-promoting bioactive ingredients. Peptides with anti-inflammatory, immunomodulatory, and neuroprotective effects found in sheep milk contribute to its potential in supporting the treatment of metabolic diseases, cancer, and neurodegenerative conditions [4]. The presence of bioactive compounds such as conjugated linoleic acid (CLA) and orotic acid in sheep milk has been associated with the prevention of type 2 diabetes, Alzheimer’s disease, and certain cancers. Additionally, sheep milk may be better tolerated by individuals with lactose intolerance or hyper-sensitivity to cow milk proteins. This makes sheep milk and its products a suitable alternative for individuals who experience adverse reactions to cow milk [8,9,10,11].

In the face of an aging society and the increasing incidence of metabolic and lifestyle diseases, it is necessary to search for effective and safe preventive and therapeutic agents. Natural substances contained in sheep milk can play a key role in complementing and supporting conventional treatment methods, while offering lower healthcare costs and better quality of life for patients. Despite the known health-promoting properties of sheep milk, there is a lack of comprehensive research on their practical application in modern medicine and pharmacy. Understanding and using the full potential of the bioactive components of sheep milk can lead to the development of new therapies and health products that will be effective, safe, and available to a wide range of patients. The dietary supplement market is seeing growing interest in products containing isolated ingredients from sheep milk, such as whey proteins and colostrum. Sheep colostrum, secreted first after birth, is rich in immunoglobulins, growth factors, and other ingredients that support the immune system. Supplements and cosmetics containing sheep colostrum are used to strengthen immunity, improve intestinal function, and support the regeneration of the body [12]. Due to their nutritional and bioactive properties, supplements with isolated ingredients from sheep milk, mainly proteins, are a valuable dietary supplement, especially for people looking for an alternative to products of cow origin.

**Figure 1:**
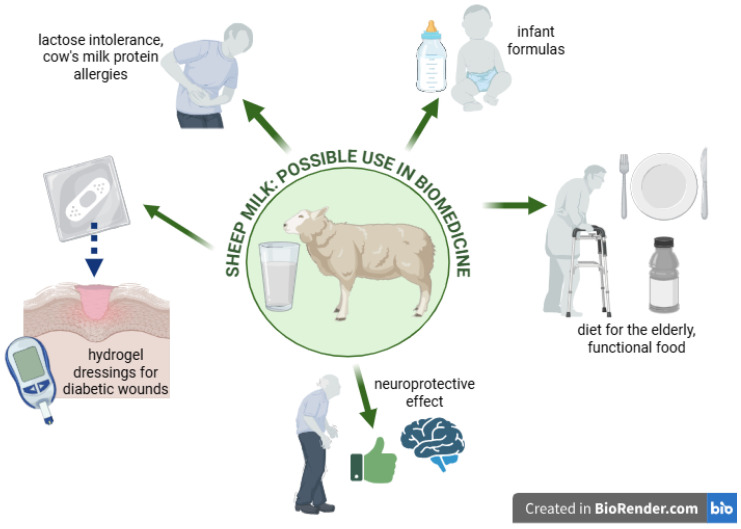
Graphical presentation of the issues discussed in the article. Created in Biorender. Piotr Szatkowski. (2025) https://BioRender.com/.

In summary, sheep milk and its bioactive compounds offer significant potential in various therapeutic applications, including wound healing, functional nutrition, and the management of chronic diseases (Figure 1). Ongoing research continues to explore and expand the clinical uses of sheep milk, underscoring its importance in modern biomedical and nutritional sciences. The aim of this review is to collect and discuss current scientific reports on bioactive components of sheep milk and to assess their potential use in biomedicine. In addition, the potential applications of these components in functional products, dietary supplements, and biomedical materials are discussed. This review also aims to indicate areas requiring further research in the context of the use of bioactive components of sheep milk in medicine and dietetics.

## 2. Bioactive Substances of Sheep Milk in Biomedicine

### 2.1. Proteins

An important component of milk is protein (Table 1), which not only serves as a nutrient but can support bodily functions. Bioactive peptides are defined as peptide sequences in proteins that exert beneficial effects on body functions and positively influence human health [13]. These peptides can regulate many important processes in the body, e.g., antimicrobial, anticoagulant, or immunomodulatory effects [6]. Therefore, interest in these substances as nutraceuticals has increased in recent years [14,15]. Bioactive peptides derived from milk are formed by proteolysis of casein (α-, β-, γ-, and κ-casein) and whey proteins (β-lactoglobulin, α-lactalbumin, serum albumin, immunoglobulins, glycomacropeptides, lactoferrin, lactoperoxidase, and lysozyme) [16]. Sheep milk is the richest source of whey protein and casein [17,18]. Sheep milk’s casein molecules account for about 80% of the total milk protein, compared to 50% in mare milk and less than 50% of the total protein in human milk [19].

The high nutritional value of sheep milk is also related to its proline content, which affects hemoglobin production. Additionally, research by Singh et al. (2017) showed an indirect effect of proline on the proliferation of MCF-7 and MDA-MB-231 breast cancer cells [21]. Sheep milk proteins contain the highest amount of proline and its derivative, hydroxyproline. The proline content in whey milk proteins is as follows: sheep milk 102 mg/g, goat milk 67 mg/g, and cow milk 69 mg/g [22] (Table 2). A study by Oguz et al. (2020) showed that a proline derivative (calixarene l-proline) exhibits selective cytotoxic potential against human DLD-1 colon cancer cells and A549 lung cancer cells [23].

Another important protein contained in sheep milk that belongs to bioactive substances and can improve health is lactoferrin. Sheep milk is extremely rich in lactoferrin (0.7–0.9 g/L) compared to cow milk (0.02–0.5 g/L) [28] (Table 2). Lactoferrin has the ability to decrease the cellular receptor efficiency of pathogenic microorganisms and their hosts [29]. In addition, it also inhibits granulopoiesis and increases natural killer (NK) cell activity [1]. Studies have shown that bioactive peptides from sheep milk play a key role in controlling type 2 diabetes through mechanisms such as satiety response, lowering blood glucose levels and increasing insulin uptake, regulating incretin hormones, and reducing digestive enzyme activity [30,31,32,33]. One of the most important strategies for the treatment of type 2 diabetes is to reduce or inhibit the activity of dipeptidyl peptidase enzymes (DPP-4) and α-glucosidase [32]. Studies show that the consumption of milk and dairy products inhibits the action of the DPP-4 enzyme, which has a positive effect on stimulating insulin secretion [32,34]. Inhibitory effects on DPP-4 and α-glucosidase have been demonstrated for milk peptides of animal species such as camels [35,36,37], horses [38], cattle [37], and sheep [38]. A study by Iram et al. (2022) showed that sheep milk proteins can serve as a potential source of dipeptidyl peptidase-IV (DPP-IV) inhibitors [39]. Products obtained by hydrolyzing lactoferrin from cow milk are capable of inhibiting the DPP-IV enzyme [31]. This suggests that sheep milk, due to its higher lactoferrin content, may have a more effective DPP-IV inhibitory effect and thus may have a much more effective effect on blood glucose regulation in diabetic patients.

### 2.2. Fatty Acids

Another important component of sheep milk is fatty acids (Table 1). Sheep milk fat contains the highest level of conjugated linoleic acid (CLA) (Table 2), but also a high amount of vaccenic acid, which is its physiological precursor [20,40,41]. The cis and trans isomers of vaccenic acid contained in milk inhibit the proliferation rate of colon cancer cells and contribute to weight loss [42]. The most relevant and functional CLA isomers that appear in the highest amounts are the cis-9, trans-11 CLA, and trans-10, cis-12 CLA isomers with anticancer and lipolytic effects [43,44,45]. In addition, biochemical studies have shown the beneficial effects of cis-9, trans-11 CLA in reducing cancer, atherosclerosis, and cholesterol-lowering processes [46,47,48]. A study by Alichanidis et al. (2016) on CLA content showed its highest content in sheep milk (0.8%), compared to goat milk (0.7%) or the most available cow milk (0.7%) [20].

Due to the highest CLA content in sheep milk and its products, these products can be used as food ingredients to support weight control and obesity prevention [49]. Numerous studies demonstrate that CLA modulates fat deposition, making it a highly relevant factor in the prevention and control of obesity [19,50,51,52]. Studies in rats with diet-induced obesity suggest that the complex of fucoxanthin and CLA can lower serum triacylglycerol, glucose, and leptin levels and exhibit anti-obesity effects [53]. This occurs by regulating the mRNA expression of enzymes associated with lipid metabolism in white adipose tissue (WAT) [53]. In addition, a study by Dahiya and Puniya (2018) on mice with diet-induced obesity showed that supplementation with CLA-enriched skimmed milk alone has anti-obesity effects [54]. CLA administered to mice at a concentration of 0.5% resulted in a 57% and 75% reduction in body weight over a period of 4–8 weeks [42]. The use of CLA isomers trans-10, cis-12 and cis-9, trans-11 by humans suffering from obesity for a period of 12 weeks resulted in a reduction in body fat [55]. Feeding people with metabolic syndrome 5 mL of milk enriched with a mixture of CLA isomers contributed to a reduction in abdominal obesity and body weight [55]. CLA, by stimulating lipolysis and reducing the concentration of the enzyme lipoprotein lipase in adipocytes, influences the body’s energy metabolism. Studies conducted on vascular stromal cells derived from human adipose tissue showed that the trans-10, cis-12 isomer of CLA attenuated lipogenesis in the cells, while the cis-9, trans-11 isomer of CLA increased triglycerides [56]. It is presumed that the reduction in adipose tissue is related to a reduction in the proliferation of fat cells, not only by stimulating their programmed death, but mainly by reducing the size of adipocytes [57]. Another mechanism of fat reduction is that CLA stimulates the action of the enzyme carnitine palmitoyltransferase I (CPTI). By stimulating CPTI, CLA causes significant consumption of fatty acids that have been released from the body’s adipose tissue [58]. Much evidence suggests that obesity and overweight are often the cause of cancers of the breast, colon, kidney, pancreas, or liver [59]. In fact, overweight and obesity are now recognized risk factors for cancer and cancer-related mortality [59].

A diet rich in CLA, most of which is found in sheep milk, may be effective against cancer [60,61,62,63]. As indicated by the research of Viladomiu et al. (2016), the two most relevant isomers of CLA: trans-10, cis-12 and cis-9, trans-11 show effective anticancer activity [64]. The cis-9, trans-11 isomer of CLA mediates anti-carcinogenic effects through apoptosis. In contrast, the trans-10, cis-12 isomer of CLA can inhibit cancer cell growth and induce cell death [65]. Therefore, the consumption of CLA may play an important role in inhibiting the growth and development of cancer cells, including breast cancer. A study by Ou et al. (2007) showed that the trans-10, cis-12 isomer of CLA induces apoptosis and arrests the G1 phase of p53-mutated TM4t mouse mammary cancer cells through the mitochondrial pathway and by targeting Bcl-2 proteins [66]. The cis-9, trans-11 isomer of CLA and the trans-10, cist-12 isomer of CLA have an inhibitory effect on the proliferation of MCF-7 human breast cancer cells [67]. Additionally, research by Bruen et al. (2017) showed that women who consume CLA-rich dairy products are less likely to develop colon cancer [68]. The most biologically active isomer of CLA is the cis-9, trans-11 isomer, which inhibits the occurrence and development of skin cancer [69]. A study by Park et al. (2010) showed that CLA supplementation accelerates skin wound healing by regulating antioxidant and anti-inflammatory functions [70]. The cis-9, trans-11 isomers of CLA and the trans-10, cis-12 isomers of CLA have effects on specific T-lymphocyte populations and immunoglobulin subclasses [71]. As shown in the study by Bassaganya-Riera et al. (2012), CLA modulates the immune response by inhibiting the ability of peripheral blood T lymphocytes to produce pro-inflammatory cytokines [71].

A major health problem in society is cardiovascular-related diseases. Particular attention should be paid to arterial disease, commonly known as atherosclerosis, which leads to partial or even complete closure of the vessel lumen, resulting from rupture of atherosclerotic plaques and the formation of thrombi on their surface [72]. The cause of this disease is too much fat intake with food, especially the oxidation of cholesterol from animal products [73]. Symptoms arising from atherosclerotic lesions are associated with endothelial dysfunction, which is due to the presence of diabetes, high LDL cholesterol fraction, infections, or hyperhomocysteinemia. Sheep milk CLA has a beneficial effect in reducing not only the LDL and triglyceride fractions, but also total cholesterol, counteracts hypertriglyceridaemia, and regulates the blood lipid profile. A study by DeClercq et al. (2012) showed that the trans-10,cis-12 isomer of CLA can reduce obesity-related hypertension as a result of increased adiponectin and endothelial nitric oxide synthase activity [74]. In patients at risk of cardiovascular disease, consumption of cheese naturally enriched in CLA significantly improves the plasma lipid profile and regulates inflammation by increasing high-density lipoprotein (HDL) cholesterol (high-density lipoprotein fraction cholesterol) and decreasing blood levels of C-reactive protein (CRP) [75].

### 2.3. Polar Lipids

Among the components of milk fat, polar lipids such as sphingomyelin, phosphatidylcholine, and phosphatidylethanolamine deserve special attention. The polar lipid fractions of sheep milk are involved in several physiological processes and have functional and nutritional properties. Sheep milk fat is rich in polar lipids (Table 2), which have anticoagulant effects, lower atherogenic cholesterol, and reduce atherosclerosis, modulate gut microflora, and reduce inflammation in the liver and blood serum [2,3,76]. Platelet-activating factor (PAF) is an essential mediator of inflammatory phospholipids. The presence of PAF inhibitors in foods enhances their nutritional value for protection against cardiovascular disease. Studies in mice with the low-density lipoprotein (LDL) receptor turned off have shown that the addition of milk to feed reduces the development of atherosclerosis compared to animals fed a high-fat diet without milk [3]. Importantly, the fermentation process enhances the anticoagulant properties of polar lipids against PAF inhibitors, and yogurts made from sheep and goat milk contain numerous active PAF inhibitors [77,78]. The anticoagulant effect of polar lipids has also been demonstrated in traditional Greek cheeses made from sheep milk, such as Ladotyri and Kefalotyri [3,79].

### 2.4. Orotic Acid

Orotic acid is another important bioactive component of sheep milk. Orotic acid is an intermediate product in the pyrimidine biosynthesis pathway and is necessary for the regulation of genes that are particularly important for cell, tissue, and organ development [80]. Sheep milk contains more orotic acid (20–400 mg/mL) than human milk (less than 2 mg/L) and cow milk (20–100 mg/mL) [24] (Table 2). A diet rich in this acid may be important for patients who are at higher risk of developing lifestyle-related cardiovascular disorders [81]. Acid and its magnesium salt (magnesium orotate) increase the energy status of the muscle after infarction. In turn, magnesium orotate increases the exercise tolerance of people with coronary artery disease. Orotic acid stimulates the production of erythrocytes and thrombocytes, thereby protecting the body from ischaemic stress. In addition, it helps with cardiomyopathy and delays the symptoms of aging. It can also increase cardiac contractility and prevent the accumulation of cholesterol plaques in blood vessels and exhibit anti-atherosclerotic effects [81]. Orotic acid is also of great interest in medicine and the design of new anticancer drugs. Numerous studies have shown that platinum, palladium, and zinc orotates induce anticancer activity against MCF-7 (breast), HEK-293 (kidney), PC-3 (prostate), HCT-15 (colon), and HepG2 (liver) cancer cell lines [82,83]. A study by Marynowicz et al. (2023) showed that orotic acid can also be considered a promising therapeutic strategy for ovarian granulosa tumors [83]. Orotic acid acts selectively to induce apoptotic death of ovarian granulosa tumor cells, without negatively affecting normal granulosa cells. The anti-tumor properties of orotic acid are probably related to the increased activity of caspase 3/7 [84]. This is a naturally occurring compound in milk that is currently of great interest to researchers due to its potential to improve cellular energy efficiency.

### 2.5. Insulin

Insulin is an important metabolic hormone that is responsible for regulating blood glucose levels and is the basis for the treatment of type 1 diabetes [85]. Insulin has been found to be involved in promoting fat deposition and regulating hepatic production of insulin-like growth factor 1 (IGF-I 1) and insulin-like growth factor binding protein type 1 (IGFBP-1) [86]. There is ample evidence that insulin and related hormones play an important role in development and metabolism, and that abnormalities in insulin secretion and uptake can lead to hyperglycaemia and diabetes [87].

Diabetes mellitus, especially type 2, is a major global health problem with an increasing prevalence worldwide [4]. In this aspect, bioactive peptides that can be used as nutraceuticals for the prevention of metabolic disorders in humans deserve special attention [88]. Bioactive peptides may act as agents against type 2 diabetes due to their ability to inhibit the enzymatic activity of dipeptidyl peptidase-IV (DPP-IV), α-amylase, and α-glucosidase [33]. A study by Han et al. (2022) demonstrated the positive effects of ruminant milk in the prevention of diabetes and reduction in insulin resistance [88]. Numerous studies support the effectiveness of camel milk in the treatment of diabetes due to its high levels of insulin, approximately 52–59 units/liter, and other insulin-like substances [35,89,90]. Sheep milk is also a promising source of insulin, antidiabetic, and antihypertensive peptides [39,91,92]. Recent reports by Zhang et al. (2024) confirmed that sheep milk, compared to cow milk, is a potential antidiabetic agent [92]. The study showed that supplementation with sheep milk alleviated insulin resistance in mice fed a high-fat diet. In contrast, treatment with cow milk exacerbated systemic insulin resistance induced by a high-fat diet [92]. Studies confirm that whey protein consumption can significantly improve glucose tolerance in healthy individuals. Sheep milk contains more whey protein than cow or goat milk, which translates into its better digestibility and nutritional value [17]. Consuming whey protein, especially from sheep milk, can be beneficial in regulating blood glucose levels in healthy individuals. Due to its high content of whey protein and the presence of bioactive peptides, sheep milk is a valuable element of a diet supporting glucose metabolism.

### 2.6. Selected Antioxidant Substances and Minerals

An extremely important feature of sheep milk is its high content of many antioxidant substances, including vitamins A, E, and C. Sheep milk is also a valuable source of minerals such as iron, copper, and zinc (Table 2) [4,18]. Vitamin A is crucial for the proper functioning of the immune system, vision, and epithelial cell differentiation processes [93]. Vitamin A, especially in the form of retinol, is important for skin health. It supports the development of skin cells, stimulates collagen production, and can help alleviate psoriasis and counteract the skin aging process. Vitamin A stimulates the process of angiogenesis and collagen synthesis, as well as the growth of epithelium, fibroblasts, and granulation tissue [4]. Due to its antioxidant and skin care properties, vitamin A is successfully used in cosmetics and pharmacy [18,94,95]. Its antioxidant effect helps neutralize free radicals, which may contribute to reducing the risk of developing many diseases, including cancer [18,55]. Moreover, vitamin A participates in maintaining a healthy immune system, among others, by participating in the production of antibodies [18]. Moreover, vitamin A is essential for the proper growth and development of bones and maintaining the health of cartilage and joints, which is why it plays a key element in the diet of children and the elderly [95]. However, its effect is complex and requires proper balancing. Sheep milk, due to the highest content of vitamin A and retinol (Table 2), compared to the milk of other ruminants, can be its key source in the design of functional food for patients with special needs.

Sheep milk is characterized by the highest content of vitamin E (tocopherol) (Table 2). This vitamin acts as a strong antioxidant, protecting cells from oxidative damage [18,96]. It also stimulates the functioning of the immune system. Vitamin E is used in the treatment of skin diseases and in cosmetology due to its regenerative properties and its ability to delay skin aging processes [40,97]. Therefore, dressing materials and preparations for use on the skin and wounds containing sheep milk (as a natural, rich source of vitamin E) may be characterized by high biological activity and therapeutic efficacy. Additionally, studies show that vitamin E reduces the risk of neurodegenerative diseases such as dementia or Parkinson’s disease [18,97].

Vitamin C (ascorbic acid) is essential for collagen synthesis, which is crucial for the health of the skin, blood vessels, and wound healing [98]. Moreover, vitamin C supports ceramide synthesis, fibroblast maturation, and the angiogenesis process, which strengthens the lipid barrier of the epidermis and protects the skin from external factors [4]. Vitamin C also plays a role in iron absorption, which is crucial for protection against infections [98]. It acts as an antioxidant, supporting the immune system and protecting cells from oxidative stress [18,97].

The numerous minerals contained in sheep milk contribute to many physiological functions, forming essential parts of many enzymes, and have biological significance for the homeostasis of the body [99]. Minerals such as iron, zinc, and copper in the milk of ruminants, including sheep, occur mainly in the form bound to casein, which affects their bioavailability and technological properties of milk. Casein, the main protein of sheep milk, forms micelles that bind these microelements, stabilizing them and affecting their solubility and bioavailability [97].

Iron, zinc, and copper play a key role in maintaining skin health and the wound healing process [4,99]. Iron is involved in oxygen transport and collagen synthesis, zinc regulates the immune response and supports tissue regeneration, and copper is involved in the formation of collagen and elastin fibers and stimulates angiogenesis, which together accelerate the reconstruction of damaged skin structures [18,97]. Studies indicate that an appropriate level of zinc may reduce the risk of neurodegenerative diseases such as Alzheimer’s disease or Parkinson’s disease [100]. As a result, sheep milk can be a valuable element of a diet supporting brain health and may be particularly beneficial for the elderly.

The highest content of these bioactive antioxidant substances and minerals in sheep milk (Table 2) may effectively affect the significant functionality and effectiveness of many biomedical preparations—dietary supplements, special foods for the elderly and children, or dressing materials for use on wounds.

## 3. Novel Potential Applications of Sheep Milk

**Figure 2:**
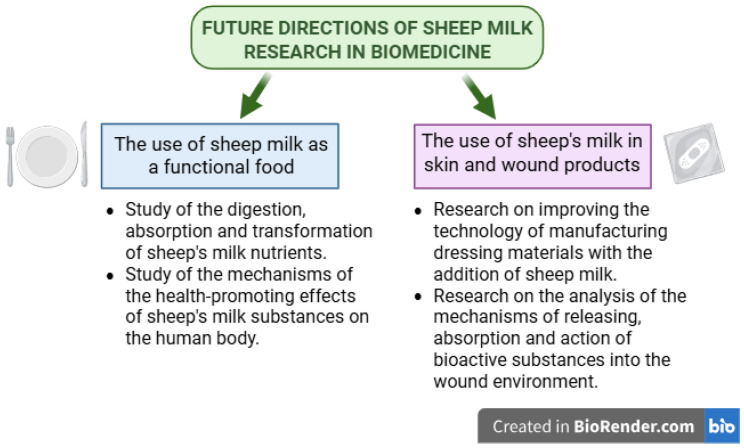
Future directions of sheep milk research in biomedicine. Created in Biorender. Piotr Szatkowski. (2025) https://BioRender.com/.

Sheep milk, traditionally used in cheese production, is gaining new importance in many areas of biomedicine. Sheep milk contains numerous bioactive substances with health-promoting effects and is characterized by high technological usefulness [98]. For this reason, it can be successfully used as an element in composite biomedical materials such as hydrogel wound dressings or dietary supplements. Furthermore, the processing of this milk often enhances its health-promoting properties [2]. Thanks to its rich chemical composition, sheep milk can be used as an effective alternative to cow milk in the context of feeding both infants and the elderly. Furthermore, sheep milk is beginning to play an increasingly important role in the functional food market for people with lactose intolerance or allergies to cow milk proteins. More and more research is devoted to the bioactive substances of sheep milk in the diet of people with neurodegenerative diseases such as dementia or Alzheimer’s disease [96]. Due to the enormous potential of the possible use of sheep milk in these areas of biomedicine, more research should be carried out, including in vitro, in vivo, and clinical studies. Future research directions should include, first of all, a comprehensive study of the mechanisms of action of individual bioactive substances. Research on hydrogel dressings from sheep milk currently focuses on refining the technology of manufacturing biocomposite materials with the addition of milk. An important issue is to investigate how substances are released from the material into the wound environment and how they support the process of tissue regeneration. In the case of research on sheep milk as a key ingredient in functional food preparations, it is important to thoroughly investigate the process of digestion, absorption, and transformation of nutrients and the mechanisms of their health-promoting effects on the human body. These studies would allow for a clear and comprehensive assessment of the possibilities of using sheep milk in biomedical fields (Figure 2).

### 3.1. Potential Applications of Dressing Biomaterials Enriched with Sheep Milk Bioactive Substances

Recently, hydrogel dressings have become the subject of much biomedical research and have gained much interest among consumers. This is due to the many bioactive properties of hydrogel materials. Hydrogel dressings are made from natural and biodegradable materials. They are characterized by high biocompatibility and lack of toxicity, while providing an appropriate moist environment to promote the wound healing process [98]. Furthermore, hydrogel dressings can be enriched with natural bioactive substances of plant or animal origin. Sheep milk, due to its high content of numerous bioactive substances such as proline, lactoferrin, or conjugated linoleic acid (CLA), among others, can be used in wound treatment as a targeted therapeutic system. Proline plays a key role in the synthesis of arginine and initiates the synthesis of proteins, especially collagen. Therefore, it is of particular importance in the wound healing process. It also influences the immune response, has an antibacterial effect, and has antioxidant properties in the wound environment. Lactoferrin supports many biological processes involved in wound healing. It exhibits anti-inflammatory effects, directly promotes granulation tissue formation, stimulates proliferation and migration of fibroblasts and keratinocytes, and enhances collagen and hyaluronate synthesis. It has a high affinity for iron ions [101]. It also has the ability to decrease the cellular receptor efficiency of pathogenic microorganisms and their hosts [29]. This is particularly important to provide a suitable environment for regenerating skin tissue and to accelerate the healing process. Sheep milk, due to its highest lactoferrin content, reduces autoimmune inflammatory processes and has a protective effect against bacterial and viral infections [102,103]. The development of novel wound dressings containing natural ingredients with highly effective antimicrobial and wound-healing properties has been the subject of much recent research. Wang et al. (2022) synthesized a hydrogel containing lactoferrin and lithium magnesium silicate that effectively promotes wound healing [104].

As reported by Mansour et al. (2015), a cream containing 25% sheep milk fat globules accelerates burn healing and reduces inflammation, accelerates wound contraction, minimizes scarring, and improves burn wound healing [105]. Among the most abundant bioactive sheep milk lipids, CLA deserves special attention [106]. Its most biologically active CLA isomer is cis-9, trans-11, inhibits the occurrence and development of skin cancer [69]. A study by Park et al. (2010) showed that CLA supplementation accelerates skin wound healing by regulating antioxidant and anti-inflammatory functions [70]. This provides an opportunity to investigate the surface effects of CLA on wound healing [98].

The lifetime risk of ulcer formation in a diabetic patient is 12–25%, and patients with diabetes are 30–40 times more likely to have an amputation than those without diabetes [98]. There is a significant increase in the number of people with obesity and diabetes, leading to a significant increase in the incidence of chronic wounds. Therefore, the development of novel hydrogel wound dressings with properties to accelerate healing has been the subject of much recent research [104]. This represents a huge opportunity for improving the comfort of patients with difficult-to-heal wounds. Recent self-reports indicate the high therapeutic potential of hydrogel dressing materials to support the treatment of wounds of various origins, including diabetic wounds [4,107].

### 3.2. Sheep Milk as a Functional Alternative for the Development of a Young Organism

The bioactive and nutritional content of milk is of great importance and influences the development of the young organism. A significant number of bioactive substances pass from the mother’s blood into the milk; others are synthesized in the mammary gland. Interestingly, some hormones (e.g., prolactin) and bioactive molecules are both synthesized in the mammary gland and transported from the mother’s bloodstream. They all then enter the newborn’s bloodstream through the mucous membranes of the stomach and intestines, interacting with specific receptors [108]. It is possible that in the neonate, these substances play a role in the synthesis of its own hormones and growth factors. These, in turn, act comprehensively, influencing the development of the structure and function of the individual organs of a young organism [109,110]. Many of the previously mentioned milk nutrients are increasingly being studied because of their role in the brain development of the young organism [111].

Breast milk is the optimal source of nutrition; however, modified milk is an alternative or complementary solution in situations where breast milk is unavailable or limited in quantity [112]. The vast majority of infant formulas are produced using ingredients derived from cow milk [113]. Claeys et al. (2014) conducted a study in which they compared horse, donkey, and sheep milk with human milk and indicated that sheep milk may be a suitable alternative to breast milk and formula milk for infants [60]. Countries such as China and New Zealand have recently been turning their attention to the use of sheep milk for the production of milk replacers for infants [95]. A study by Jena et al. (2022) showed that feeding sheep milk to piglets in the early postnatal period has a beneficial effect on brain development [112]. In contrast, a study by Lai et al. (2023) showed that formula made from sheep milk can be safely used in the feeding of newborn piglets aged 0–6 and 6–12 months [94]. The interest in non-cow milk is also due to the higher prevalence of allergies after consuming cow milk or any of its components [9,114]. Many infants and children under three years of age, but also adults, show allergic reactions to cow milk but not to goat’s or sheep milk [8,9,10,11]. Therefore, sheep milk may play a key role in people who are allergic to cow milk. A study by Masoodi and Shafi (2010) showed that the αS1 and αS2 casein protein sequences in goats and sheep show at least 99% similarity between each other, whereas they differ significantly from the αS1 and αS2 protein sequences of cow milk [9]. In addition, specific antibodies in milk-sensitized individuals, namely immunoglobulin E, (IgE) poorly recognize protein fractions of αS1, αS2 caseins from goat and sheep milk, but not cow milk [115].

Milk protein allergy and lactose intolerance are two different conditions associated with milk consumption, which are often confused due to similar symptoms, such as diarrhea, flatulence, or abdominal pain [116]. Milk protein allergy is manifested by an immunological reaction to milk proteins and requires the complete elimination of milk from the diet [117,118]. Lactose intolerance, on the other hand, results from a deficiency of the enzyme lactase, which leads to the fermentation of undigested lactose in the large intestine and gastrointestinal symptoms [118]. Sheep milk is a nutritionally rich alternative to cow milk that can be digested differently due to its unique composition and physicochemical properties [119]. Compared to cow milk, the lactose content of sheep milk is about the same, while the fat and protein levels are much higher [120]. This means that the lactose content of sheep milk is actually lower relative to the total solids content compared to the total solids content of cow milk [120]. Diagnosis of lactose intolerance includes tests such as the hydrogen breath test, lactose tolerance test, and genetic testing. Furthermore, studies show that the digestive discomfort associated with milk intolerance is complex and is influenced by more components of milk than just lactose [119]. Casein constitutes about 80% of the total protein in milk, making it the main protein component in milk [5]. Its basic functions are the transport of calcium and phosphate ions, which are crucial for bone mineralization in young individuals, and the supply of essential amino acids to the body [121]. Casein includes α S1-casein, α S2-casein, β-casein, and k-casein. Sheep casein contains 45% β-casein, represented by two phosphorylated forms, β1-casein and β2-casein, which have a similar amino acid composition to bovine β-caseins and have a significant effect on milk protein [122]. Studies have shown that milk containing A2 β-casein causes fewer and milder symptoms of lactose intolerance [123].

### 3.3. Sheep Milk as a Functional Food for the Elderly

Sheep milk, thanks to its high content of bioactive substances such as lactoferrin, lysozyme, immunoglobulins, and bioactive peptides with anti-inflammatory and antioxidant effects, is a valuable ingredient in functional foods, particularly suitable for the elderly [28,124]. These constituents exhibit a broad spectrum of biological activity, including immunomodulatory properties, neuroprotective properties, and the ability to reduce oxidative stress, making them important in the prevention of age-related diseases [96]. An important feature of sheep milk is the significantly higher protein content, mainly whey protein, than in cow milk [17,18,27]. This is particularly important in the context of preventing the loss of muscle mass and strength that accompanies aging [125,126]. High biological value protein provided in the diet supports tissue regeneration, maintenance of muscle mass, and overall metabolic performance in older adults. Additionally, fermented sheep milk products such as yogurts and cheeses can support the balance of the gut microbiota, the proper functioning of which is crucial for health in old age [27]. There is growing interest in the possibility of developing functional foods based on sheep milk, dedicated to consumers with specific nutritional needs [127]. Moreover, there has recently been a growing body of research indicating a link between diet and cognitive function and the risk of dementia. Scientific evidence suggests a direct link between diet and changes in brain structure and activity [128].

There are currently no studies that directly evaluate the effects of sheep milk and its products, such as yogurt or cheese, on cognitive function in older adults or patients with Alzheimer’s disease. However, there are studies examining the general effects of dairy consumption on cognitive function in the elderly population. For example, a study by Han et al. (2024) showed that the moderate consumption of fermented dairy products, such as yogurt and cheese, was associated with better performance on tests of executive function and verbal fluency in older adults [129]. A study by Kaura et al. (2022) showed that fresh goat milk given for 10 days to young and old mice improved their memory in various behavioral tests and reversed ethanol- and scopolamine-induced amnesia [100]. A decrease in acetylcholinesterase activity, cholesterol levels, and oxidative stress was also observed, while glutathione levels increased, indicating the strong antioxidant properties of goat milk [100]. Preliminary studies in patients with mild dementia suggest that goat milk, either alone or in combination with donepezil, may support cognitive function [100]. Another study by Safdar et al. (2020) demonstrated the effectiveness of goat milk supplementation on memory performance in a D-galactose-induced aging rat model [130]. Safdar et al. (2020) suggest that one factor influencing these milk abilities is the high conjugated linoleic acid (CLA) content in goat milk [130]. Studies in rats have shown that a maternal diet rich in CLA promoted better brain development and cognitive function in offspring [131]. This effect is attributed to the strong antioxidant properties of CLA, which can protect neurons from oxidative stress, a known factor that accelerates brain aging and impairs memory [130]. Sheep milk, due to its higher CLA content compared to the milk of other ruminants, indicates the effectiveness of sheep milk in improving memory and may therefore be useful in protecting against age-related memory deficits.

An adequate diet may act as a preventive factor for many chronic, metabolic, or neurodegenerative diseases, including Alzheimer’s disease [132]. Lactoferrin is a multifunctional glycoprotein characterized by a high affinity for iron ions, exhibiting a range of biological properties, including antimicrobial, anti-inflammatory, and immunomodulatory [101]. Its content in sheep milk is significantly higher (0.7–0.9 g/L) compared to cow milk (0.02–0.5 g/L), making sheep milk a particularly interesting source of this bioactive substance [28]. It is noteworthy that abnormalities in iron metabolism, including iron synthesis and transport, are recognized as one of the important markers of early-stage Alzheimer’s disease. Studies show that patients with Alzheimer’s disease have elevated levels of this element in the cerebral cortex, subcortex, and white matter of the brain [133]. Lactoferrin decreases the production of reactive oxygen species in the hippocampus, leading to the alleviation of oxidative stress and inflammatory reactions and a reduction in iron deposits [133,134]. Lactoferrin administration reduces memory impairment and Aβ aggregation in a mouse model of Alzheimer’s disease and improves cognitive abilities in a naturally aging C57/BL6J mouse model [134,135]. Lactoferrin administered to patients with Alzheimer’s disease attenuates cognitive decline by modulating the p-Akt/PTEN pathway, thereby affecting inflammation and oxidative stress [124]. Lactoferrin has the ability to penetrate the brain parenchyma and overcome the blood–brain barrier, which allows only a few types of molecules to pass from the circulation into the central nervous system [136]. Lactoferrin crosses the blood–brain barrier mainly through receptor-mediated transcytosis [137]. This process involves the binding of lactoferrin to its specific receptors on the surface of cerebral vascular endothelial cells, neurons, and glial cells, which initiates its internalization and transport through the cells to the brain [29]. Due to these properties, lactoferrin is being investigated as a potential drug carrier in the treatment of neurodegenerative diseases such as Alzheimer’s disease, enabling the delivery of therapeutic substances to the brain.

In addition, lactoferrin can also form micelles with other biologically active molecules, thereby precisely delivering molecules to the brain. This may be of particular importance in the treatment of many neurodegenerative diseases, including Alzheimer’s disease, where a significant problem is the low efficacy of administered drugs due to the presence of the blood–brain barrier. A study by Agwa et al. (2020) showed that micelles of CLA combined with lactoferrin have active penetration in brain tissue, which contributes to increased cognitive abilities and reduces oxidative stress and inflammation [138]. This creates a huge opportunity in the medical field for the use of lactoferrin as an advanced drug delivery system.

Parkinson’s disease is the second most common neurodegenerative disorder in humans, and is characterized by, among other things, increased oxidative stress and progressive neuronal degradation [139]. Studies by Ubaid et al. (2020) show that α-lactalbumin may be of particular importance in the course of Parkinson’s disease [140]. Studies in a cellular model of Parkinson’s disease showed that treatment with a complex of camelina α-lactalbumin and oleic acid reduced oxidative stress and increased cell viability [140]. Sheep milk contains more α-lactalbumin (1.2–2.6 g/L) than cow milk (1–1.5 g/L) and therefore may be a good alternative as a nutraceutical supplement to improve neurological function in elderly people at increased risk of Parkinson’s disease [28,141].

## 4. Conclusions

Sheep milk is gaining popularity among consumers, especially in the form of cheese and yogurt, which is in line with the growing interest in functional food and the return to traditionally produced high-quality dairy products. Thanks to its unique physicochemical and nutritional properties, sheep milk is a valuable source of natural bioactive substances such as proteins, peptides, fatty acids, vitamins, and minerals. Moreover, it contains high levels of conjugated linoleic acid (CLA), known for its anti-inflammatory and anticancer properties, as well as vitamins A and E, which support immune function and cellular health. The proteins in sheep milk have been associated with various physiological effects, such as antihypertensive, antimicrobial, antioxidant, and immunomodulatory activities. Moreover, compared to cow milk, sheep milk contains a higher concentration of lactoferrin, which has antibacterial properties and supports tissue regeneration, which is extremely important in the treatment of difficult-to-heal wounds. The mentioned bioactive substances of milk have immunomodulatory, anti-inflammatory, and neuroprotective effects, making sheep milk a promising ingredient in the prevention and support of the treatment of lifestyle diseases, including type 2 diabetes, circulatory system diseases, cancers, and neurodegenerative diseases such as Alzheimer’s disease. Compounds present in sheep milk, such as proline, lactoferrin, orotic acid, and conjugated linoleic acid (CLA), which support cognitive functions and tissue regeneration, are particularly important in the context of an aging society. Due to its health-promoting properties, sheep milk is used in the nutrition of the elderly and infants, especially in cases of allergy to cow milk proteins. All this makes sheep milk have great potential in the development of functional foods and therapies supporting the treatment of many chronic and degenerative diseases.

## Figures and Tables

**Table 1:** Chemical composition of sheep, cow, and goat milk [20].

Chemical Composition	Sheep Milk	Cow Milk	Goat Milk
Dry matter (g/kg)	178	127	132
Protein (g/kg)	57	34	36
Fat (g/kg)	74	38	43
Lactose (g/kg)	48	48	44

**Table 2:** Comparison of the content of bioactive substances in sheep, cow, and goat milk.

Bioactive Substances	Sheep Milk	Cow Milk	Goat Milk
Proline (in whey milk proteins) (mg/g) [22]	102	69	-
Lactoferrin (g/L) [20]	0.7–0.9	0.02–0.5	0.02–0.3
Total casein (g/kg) [20]	48	26	30
Lactose (g/kg) [20]	48	48	44
Conjugated linoleic acid (CLA) (% of total fatty acids) [20]	0.3–1.8	0.3–1.6	0.7
Orotic acid (mg/L)	20–400 [24]	20–100 [24]	20–400 [24]
Total polar lipids (% of total fatty acids)	0.39 [25]	0.36 [26]	-
Iron (mg/100 g) [27]	0.1	0.1	0.06
Zinc (mg/100 g) [27]	0.6	0.4	0.43
Cooper (mg/100 g) [27]	trace	0.04	0.1
Retinol (μg/100 g) [27]	64	35	0.04
Vitamin A (μgRE/100 g) [27]	64	37	54.32
Vitamin E (mg/100 g) [27]	0.11	0.08	0.04
Vitamin C (μg/100 g) [27]	4.6	0.15	0.2

## Data Availability

Data are contained within the article.
